# Early Autism Screening: A Comprehensive Review

**DOI:** 10.3390/ijerph16183502

**Published:** 2019-09-19

**Authors:** Fadi Thabtah, David Peebles

**Affiliations:** Department of Psychology, School of Human and Health Sciences, University of Huddersfield, Huddersfield HD1 3DH, UK; d.peebles@hud.ac.uk

**Keywords:** autism spectrum disorder, behaviour science, DSM-5, screening methods, clinical diagnosis, child psychology, machine learning, public health

## Abstract

Autistic spectrum disorder (ASD) refers to a neurodevelopmental condition associated with verbal and nonverbal communication, social interactions, and behavioural complications that is becoming increasingly common in many parts of the globe. Identifying individuals on the spectrum has remained a lengthy process for the past few decades due to the fact that some individuals diagnosed with ASD exhibit exceptional skills in areas such as mathematics, arts, and music among others. To improve the accuracy and reliability of autism diagnoses, many scholars have developed pre-diagnosis screening methods to help identify autistic behaviours at an early stage, speed up the clinical diagnosis referral process, and improve the understanding of ASD for the different stakeholders involved, such as parents, caregivers, teachers, and family members. However, the functionality and reliability of those screening tools vary according to different research studies and some have remained questionable. This study evaluates and critically analyses 37 different ASD screening tools in order to identify possible areas that need to be addressed through further development and innovation. More importantly, different criteria associated with existing screening tools, such as accessibility, the fulfilment of *Diagnostic and Statistical Manual of Mental Disorders* (*DSM-5*) specifications, comprehensibility among the target audience, performance (specifically sensitivity, specificity, and accuracy), web and mobile availability, and popularity have been investigated.

## 1. Introduction 

Autism spectrum disorder (ASD), a key topic in today’s clinical industry throughout the globe, refers to a pervasive developmental disorder that hinders an individual’s skills in socialisation, creates repetitive behaviours, and impacts expressive or verbal communication with disruptions ranging from moderate to severe [1]. The symptoms of autism are more visible and easy to identify in children two to three years of age. According to [2], one out of every 68 children has autism. Consequently, various screening methods have been developed by leading medical experts and psychiatrists across the world seeking to identify autistic traits in their primitive stage so as to readily provide the necessary medications [3]. 

Diagnosing ASD is a challenging task since there are currently multiple clinical techniques available, with most typically involving long-term observation and in-depth evaluation by licensed healthcare professionals [4,5,6]. Conventional diagnostic procedures for ASD require medical professionals to conduct a clinical assessment of the patient’s developmental age based on a variety of domains (e.g., behaviour excesses, communication, self-care, social skills). This widely accepted approach is referred to as clinical judgment (CJ) [7]. Until recently, most clinicians have used the *Diagnostic and Statistical Manual fourth edition* (*DSM-IV*) as the underlying criteria for diagnosing autistic behaviours [8]. The *DSM-IV* classifies autism under the category of common pervasive development disorders (PDDs). This class includes the symptoms and areas of development that need to be observed in order to identify any PDDs. The *DSM-IV* introduced four diagnostic subcategories of PDDs: Asperger disorder, pervasive development disorder—not otherwise specified (PDD-NOS), Rett’s disorder, and childhood disintegrative disorder (CDD). In 2013, the *DSM-IV* was revised, which posed a significant challenge to the way individuals are assessed as being on the ASD spectrum and necessitated refinements of existing diagnosis as well as screening techniques. 

There are many clinical and self-screening methods available to assess individuals with ASD. The most popular clinical methods include Autism Diagnostic Interview-Revised (ADI-R), Autism Diagnostic Observation Schedule (ADOS), Childhood Autism Rating Scale (CARS), Joseph Picture self-concept scale, and the social responsiveness scale [9,10,11,12]. These are clinical methods used for formal ASD diagnosis and treatment planning [13]. The techniques, like ADI-R and ADOS, have been clinically proven to be effective instruments in differentiating autism from other related developmental disorders, and having adequate validity and sensitivity [14]. However, they have been criticised for being time consuming, having long questionnaires and scoring methods, and requiring licensed clinicians and observers to administer them [15,16,17,18].

Apart from clinical diagnostic methods, there are self-administered screening instruments developed by different neuroscientists and psychologists in the autism and healthcare arena. The tools, such as Autism Spectrum Quotient (AQ), Childhood Asperger Syndrome Test (CAST), and the Modified Checklist for Autism in Toddlers (M-CHAT), which are discussed in later sections, often consist of large sets of items for discriminating the autistic behaviours from all other types of PDDs [19,20,21]. Most of these tools have been developed based on CJ methods, and have been able to present more accessible ways for users to undergo an ASD screening. Nevertheless, screening tools are not considered diagnosis methods for ASD since many of them lack the presence of a licensed clinician as well as the necessary clinical environment. In addition, the majority of these screening tools do not fully align with the new criteria for ASD developed under the *DSM-5*. Therefore, the need for revised methods that adhere to the standards of the *DSM-5* have arisen. 

There have been many studies in applied behavioural sciences that have investigated the efficiency and effectiveness in clinical environments of ASD diagnosis techniques [22,23,24,25]. However, limited studies have been carried out to identify the performance of ASD screening methods and to evaluate their merits and issues [2,26,27,28]. For instance, [26], reviewed common screening methods related to autism and only compared their performance with regard to specificity and sensitivity. A small number of details about the screening methods were provided, and important aspects such as DSM-5 fulfilment, the methods’ popularity, and their target audience were omitted. Ref. [27] reviewed early screening methods for toddlers without covering other important aspects relating to adolescents, children, and adults. They indicated that early identification of ASD traits in toddlers, 18–24 months of age, is consistent with the recommendations of the American Academy of Paediatrics. Another similar review of ASD tools for infants was conducted by [2], and showed that a two-level screening can help improve the reliability of the process. Ref. [28] conducted a systematic review of common diagnosis methods of ASD in low and middle-income countries. They revealed that because of the limited clinical resources in low-income countries, screening methods are more effective in discovering autistic traits. However, clinical diagnosis methods seem more widely utilised in middle and high-income countries. The review by [28] noted extensive variation in the design and screening mechanisms and limited population features, therefore the study’s recommendations may not be generalised. 

This article critically evaluates the available ASD screening methods in order to recognise the merits, performance issues, and shortcomings (not only in terms of sensitivity, and specificity, but also critical issues related to administration, efficiency, target audience, complexity, digital existence, and accessibility among others) for each available method. Furthermore, the screening methods that cover all target ages (toddlers, children, adolescents, and adults) are critically analysed, making this review comprehensive and applicable to the entire population of cases. For convenience, all identified methods are categorised according to their target audience, i.e., toddlers and children, adolescents and adults, and hybrids. The screening methods included in the hybrid category facilitate the screening of toddlers, children, adolescents, and adults all together.

The article consists of four main Sections. Section 2 reviews and critically analyses the ASD screening tools considered. Section 3 is devoted to a comprehensive discussion that contrasts and evaluates the identified screening tools in terms of their method of administration, accessibility, popularity, performance, and comprehensibility. Lastly, Section 4 contains suggestions for the different stakeholders involved in ASD research and summarises conclusions that abridge the findings of the study. 

## 2. Literature Review: ASD Screening Methods 

### 2.1. Toddlers and Children ASD Screening Methods

#### 2.1.1. Quantitative Checklist for Autism in Toddlers (Q-CHAT) Based on Questionnaires 

The Q-CHAT, one of the oldest methods of screening for autism, was developed by [29], as an efficient quantitative checklist to be administered by medical professionals coinciding with a report submitted by the child’s parents based on observations of the child’s behaviour. The earliest version of Q-CHAT was used to detect autism in toddlers aged between 18 and 24 months only. A screening study carried out to test the validity of Q-CHAT, based on 16,235 toddlers, revealed that the sensitivity of Q-CHAT’s initial version was as low as 38%. The M-CHAT, a modified version, was thus introduced by [19] to enhance the sensitivity of the original CHAT method. A similar screening study was conducted for M-CHAT, and it was discovered that it had higher sensitivity and specificity on the referred sample population despite those of the M-CHAT method on the over-all population remained in question. However, both the CHAT and M-CHAT consisted of over 20 Likert Scale-type questions that needed to be completed in order to assist healthcare specialists in differentiating actual cases from the controls for further referrals. The Modified Checklist for Autism in Toddlers, Revised, with Follow-Up (M-CHAT-R/F) [19] is a two-phase parent screening method to evaluate the risk of ASD. The M-CHAT-R/F can be used freely for research, educational and clinical purposes. 

The M-CHAT was later shortened to ten questions in order to make it more convenient and less time consuming for clinical and medical professionals to complete the screening process [3]. The methodology and questions were revised, retaining only the most significant items during the screening and was based on a computed discrimination index. The CHAT-23, the Chinese version of Q-CHAT, extended the screening population to toddlers aged from 16 to 30 months [30]. One of the most recent modifications, 10-Q-CHAT, is now available in many different languages, making it more accessible to healthcare professionals globally. It has proven to be an acceptable version, with 91% sensitivity and 89% specificity. The results of the CHAT method are generated based on a five-point scale (0–4), where a higher score indicates an increased possibility of positive autism symptoms [31]. 

#### 2.1.2. Autism Screening Instrument for Educational Planning—3rd Version (ASIEP-3)

The ASIEP-3 is a special tool kit, consisting of an Autism Behaviour Checklist (ABC), a sample of vocal behaviour assessment, interaction assessment, educational assessment, and prognosis learning rate developed by [32] using normed data gathered from a sample population in the United States. Designed to identify children with a high level of autistic behaviour, aged from 0–2 years and 11 months to 13 years, it also develops programmes to educate such children and to monitor their progress over time. The Autism Behaviour Checklist (ABC) is the initial screening tool used to identify autism cases and to sample vocal behaviour by measuring communication and language problems of the child (such as babbling and repetitiveness). The interaction assessment then monitors the child’s spontaneous social behaviour while educational assessment measures the functioning level of the child. The prognosis learning rate helps the user to understand the child’s progress and speed of learning. The ASIEP-3 collects the normed data by using ABC questionnaires distributed among parents, teachers, caregivers, school psychologists, and other healthcare professionals in addition to providing activities for them do with the children that identify their special needs [33]. The ASIEP-3 is more time consuming than the other methods of screening for autism, taking approximately 90–120 min to complete a single screening process, whereas methods like CHAT take only five minutes [26].

#### 2.1.3. Autism Behaviour Checklist (ABC) 

The Autism Behaviour Checklist (ABC) is a commonly used ASD screening tool with a rating scale developed by [32] as an attempt to identify children with autism. The ABC provides parents and teachers of children aged between 12 and 14 years a questionnaire to complete, consisting of 57 items, to capture the symptoms associated with the child’s behaviour. These are categorised under five different subscales: Sensory behaviour; communication and language skills; behaviour related to object use; body language; and social and adoptive behaviours. The ABC is a subtest of ASIEP’s third version and mostly consists of questions with scores ranging from 1–4 according to the level of mental or behavioural impairment of the child and based on the answers given by the parents, teachers, or the caregivers [32] The result is then analysed by well trained professionals. In the initial stage, the measures are utilised to distinguish the child’s situation from autism and other types of neurodevelopmental disorders, such as obsessive-compulsive disorders. Once the child’s autism is confirmed, further evaluations are then made to understand the areas for development required by the child [34]. According to the research by [35] which used 167 sample cases of autism, it was revealed that ABC has 77% sensitivity, 91% specificity, and 80% overall accuracy rates. 

#### 2.1.4. Screening Tool for Autism in Toddlers and Young Children (STAT)

The STAT is another interactive screening tool for differentiating children with autism from those with other developmental disorders. Dr. Stone and her team developed STAT during her service at Vanderbilt University as a lecturer of psychology and director of the university’s autism centre [36]. The STAT is administered by a trained professional examiner to children aged between 24 and 36 months who are suspected of having autism. This tool kit is designed for the use of community service providers, such as healthcare professionals, speech language pathologists, psychologists, paediatricians, preschool teachers, and social workers who by working closely with the children, are able to best assess their behavioural and communication impairments. The interactive social behaviours, such as imitation, play, reaction, and requesting, are examined directly by using the STAT tool kit. This kit includes a user manual, test protocol, scoring sheet, and simple toys like balloons, trucks, dolls, and balls. However, the kit contains 12 items and takes approximately 20 min to complete. Once all the items are completed, the administrator determines the item scores based on the child’s behaviour and responses to the different activities. The item scores can be pass, failed, or refused. This distinguishes the children with a risk of autism, and those who are identified as cases of autism are directed to the necessary healthcare professionals for further evaluation and if necessary, medication. Through an exploratory study carried out on 71 children aged from 24 to 36 months, and the observations made on their behaviours over a period of 24 months, it was discovered that STAT has 95% sensitivity and 73% specificity rates [37].

#### 2.1.5. Childhood Autism Rating Scale (CARS-2)

The CARS was developed by [9] to recognise young children with symptoms of ASD and to distinguish their severity through direct observation of the child’s functioning in responses that include smell, touch, light, sound, body use, social behaviour, verbal and nonverbal communication, and consistency of intellectual response. The initial version of CARS was focused only on children aged below 6 years, and later in the CARS- 2nd version was extended to children aged from 6 to 13 years [38]. The revised version is more responsive to high functioning individuals, leading to a higher clinical value in CARS-2 than in the CARS original version [9]. The CARS- 2 consists of three basic components: The standard version rating booklet (CARS2-ST), the high functioning version rating booklet (CARS2-HF), and a questionnaire for parents and caregivers (CARS2-QPC). The standard version rating booklet is similar to the original version of CARS and evaluates children aged below six years with an IQ lower than the estimated average IQ level in order to identify their communication impairments and the risk of having ASD. The high functioning version of the rating booklet involves evaluating children aged between 6 and 13 years with strong communication skills and a higher IQ than the estimated average IQ level. 

The questionnaire given to parents and caregivers to complete is used as a source for gathering information required for scaling CARS2-ST and CARS2-HF. Therefore, parents, teachers, and caregivers are granted access only to CARS2-QPS. Both of the other components consist of 15 items each, and are administered by specially trained professionals, evaluators, healthcare professionals, language therapists, physicians, and other experts who are familiar with ASD screening and evaluation. The CARS-HF allows the scaling of the behaviours by using a 4-point scale based on direct observations by healthcare professionals along with those made by parents, caregivers, or any other party who is familiar with the child’s behaviour. The final score is determined by the trained professionals and the severity of autistic behaviour is interpreted by using a cut-off score. The results generated by CARS2-HF are then used by psychiatrists, pathologists, and other healthcare professionals in their diagnosis and for determining what further evaluations are required in order to identify the type of medication that needs to be prescribed. School teachers need to plan and design education programmes according to the child’s needs, and other educational professionals need to make accurate placement decisions, so they too utilize the results of CARS2-HF. The results are also used by other parties who are interested in furthering research on ASD and its screening methods. The validity studies against the diagnosis made based upon CARS-2 results showed 81% sensitivity and 87% specificity rates along with a good fit with the *DSM-5* and has been recognised as one of the best measures of autistic behaviour severity [39]. 

#### 2.1.6. Childhood Asperger Syndrome Test (CAST)

The CAST is another parental or self-completion questionnaire-based autism screening tool that was developed by [22] at the Autism Research Centre of the University of Cambridge. The CAST target population is children aged between 5 and 11 years who are suspected of having Asperger syndrome or related neurodevelopmental syndromes. A questionnaire consisting of 37 different items is administered by the parents or caregivers seeking to identify the social and communication difficulties of the child which may be symptomatic of Asperger syndrome. The determined risk of having Asperger syndrome is based on a cut-off score of 15. The school-aged children who score less than this are identified as the controls and those who score above 15 are identified as ASD cases. There are two versions of CAST: CAST-1 and CAST-2 [40]. During the initial stage (CAST-1) of the pilot study, 11,635 questionnaires were distributed among the parents of children aged between 5 and 9 years old. The children who scored between 12 and 14 were invited for a full diagnosis assessment as the second stage (CAST-2). The number of responses received for CAST-2 was higher than for CAST-1, however, the accuracy of the results generated by CAST varied with the case definition and at the cut-off point of 15. The CAST showed a rate of 100% for sensitivity and 97% for specificity [41].

#### 2.1.7. Developmental Behaviour Checklist—Early Screen (DBD-ES)

Refs. [42,43] developed a questionnaire-based ASD screening method that needs to be administered by parents, teachers, caregivers, or other guardians of the child. The questions and assessment criteria have been based on research, experiments, and problems reported over a six-month period. There are a few versions of DBD available, each consisting of 96 items. The questions are designed to be answered with three basic options, ranging from 0–2, where 0 indicates “not true as far as I know”, 1 indicates ”sometimes or somewhat true”, and 2 indicates very true”. The DBD-P is designed for children aged between 4 and 18 years to identify their behavioural and emotional difficulties through a questionnaire administered by the parents or primary caregiver(s) [43]. The DBD-T is also designed for the same age category of children, but for administration by their teachers. The DBD-ES is the version developed for infants, aged from 18 to 48 months, and consists of 17 instruments that need to be answered by the parents or their caregivers [42]. The results of DBD-ES are interpreted based on an un-weighted cut-off score of 11. The DBD-ES is an acceptable parent-administered ASD screening method for identifying children with intellectual frailties, and has a sensitivity of 83% and specificity of 48% [44].

#### 2.1.8. Early Screening for Autistic Traits (ESAT)

The ESAT’s design is based on the symptoms of ASD and seeks to differentiate infants aged 0–36 months from children with other types of developmental problems. Even though it is used for screening the behaviour of toddlers, it is focused more on detecting neurodevelopmental issues in infants at the age of 14–15 months. Ref. [45] developed ESAT in 2006 as a primary ASD screening checklist. It has 14 different instruments that are focused on a child’s behaviours during playing, eye contact, joint attention, reactions, verbal and nonverbal communication, and interest in others. The checklist is undertaken by the parents or primary caregivers of the child and administered by a trained professional health consultant. All the questions given on the checklist have either yes or no response options, and the examination takes approximately 10–15 min to complete. Even though babies who score higher in ESAT are identified as more vulnerable to autism and other related developmental impairments, it does not differentiate well between the cases and controls for infants aged below 25 months. Some infants who scored less in ESAT, and recognised as the controls, later received an ASD diagnosis [46]. Therefore, the sensitivity and specificity of ESAT is still in question and follow up studies are required to develop ESAT into a more reliable ASD screening tool. 

#### 2.1.9. Pervasive Developmental Disorders Screening Test—Second Edition (PDDST-II)

The PDDST is a parent-completed questionnaire screening test developed by Dr. Bryna Seigel in 2004 to identify symptoms related to autism and other pervasive developmental disorders, such as Asperger syndrome. The initial version of PDDST was launched in 1993 and later enhanced and upgraded to PDDST-II by removing the less effective items and adding more discriminative items [47]. The PDDST-II consists of three screening stages developed based on different settings. Stage one is referred to as a primary care screener, has 22 items, and is intended to establish a primary care setting. The second stage, developmental clinic screener, consists of 14 items and is specially designed for children who are already receiving progressive services. Stage 3 is the critical stage, where toddlers aged between 18 and 48 months are identified as autistic and discounted from having other pervasive developmental disorders. This final stage is referred to as the autism clinic severity scanner. Each stage has a designated cut-off score on which the final results are evaluated. All the items are designed to be administered by parents and other caregivers through a questionnaire or interview that takes 15 min to complete and five additional minutes to calculate the score. The sensitivity and specificity vary from one stage to another depending on their cut-off scores. According to [48], for Stage 1, the primary care screener’s, sensitivity is 92% and specificity is 91% with a cut-off score of five. Stage 2 also uses a cut-off score of five to achieve sensitivity of 73% and specificity of 49%. Stage 3 uses a higher cut-off score of eight to optimise the hit rate and achieve 58% sensitivity and 60% specificity.

#### 2.1.10. Infant Toddler Check List (ICT) 

The ICT is one component of the Communication and Symbolic Behaviour Scale—Developmental Profile (CSBS-DP) established by [49] with the intention of recognising language and communication impairment in infants aged from 6 to 24 months. Even though ICT is also used widely as an ASD screening tool, the focus of ICT is on predicting language delays before the child is even starting to talk. This method is administered mainly by the parents or other caregivers who are familiar with the child’s behaviour on a daily basis. The 24 items, with Likert Scale-type responses, can be answered either in a questionnaire format or a well-explained interview format. All 24 items are subdivided into seven language predictors: Emotion and use of eye gaze; use of communication; use of gestures; use of sounds; use of words; use of objects, and understanding of words. Each subscale consists of related items, ranging from 2 to 4 points depending on the weight given to the question. It takes approximately 5 to 10 min to complete a single checklist and once it is completed the total score is calculated based on the scores gained by each of the seven clusters. The infants who score less than 10 in any of the 7 clusters are identified as a case with high probability of language and other communication delays and are also subjected to further evaluations. Empirical research shows a high validity of this method as most of the infants who scored below 10 in ITC were later diagnosed with ASD or other developmental communication impairment [49]. The sensitivity and specificity of ITC are reported as 78% and 84% respectively. The ITC is freely available on the internet and anyone can use ICT to predict ASD and non-ASD communicational difficulties in their toddlers quickly and easily. 

#### 2.1.11. Communication and Symbolic Behaviour Scales Developmental Profile (CSBS-DP)

Ref. [49] developed a quick and easily used screening tool, CSCS-DP, to identify the risk of communication and symbolic functioning disabilities in infants aged from 6 to 24 months and children whose chronological age is 5–6 years. However, their functionality is below 24 months. The CSBS-DP kit consists of seven different components that are designed to screen and evaluate infants delayed in social communication, expressive language, and symbolic functioning, as well as to monitor, assess, and document the changes in their communication functionality over time. The first step of this tool is the Infant Toddler Checklist (ITC) which consists of 24 parent-report items that are utilised to determine whether further developmental evaluations are needed for the child (as discussed above). If the results of ITC suggest that further evaluation is required, the second step is a four-page caregiver questionnaire that discusses the seven clusters of ITC in detail and takes approximately 15–20 min to complete. A behaviour sample that involves face-to-face observations regarding certain behaviours of the child is then carried out by a well-trained professional. This process involves different behavioural experiments with the child including book sharing, communicative stimulus, language and symbolic play examinations, and constructive play observations. Through the behaviour sample method, a professional observer examines 20 different behaviours of the child and marks present or absent on the worksheet. The child’s caregiver, or the parent, is then asked to compare the child’s typical behaviour with the examined behaviour, as the next step is to understand the validity of the entire experiment. Apart from these four components, the CSBS-DP kit includes a user manual which provides instructions for the administrator, a toy kit for assisting the professional examiner in observing the child’s behaviour, and a series of instructional videos to facilitate the administration process. Even though the overall sensitivity and specificity of CSBS-DP is unknown, its high validity has been proven by the many studies conducted with it over large samples of the population [50].

#### 2.1.12. The First Year Inventory (FYI)

According to many recent studies in the context of neurodevelopmental healthcare, it has been revealed that the age of one year is the most critical stage in a child’s mental and physical development, where most of the neurobiological changes emerge. Therefore, [51] developed a parent-reported ASD method to identify the risk of autism and other developmental disorders in infants at the age of 12 months that is called the First-Year Inventory (FYI). The FYI consists of 63 different items representing social communication and sensory regulation functions of the child, and are subdivided under eight precise concepts: Social affective engagement; social orienting and open communication; sensory processing; expressive communication; reactivity; repetitive behaviours, and regulatory patterns. All 63 questions are aligned with the medical definitions of autism and other neurodevelopmental disorders and have been abstracted from the knowledge gained through an extensive literature review on similar topics of interest. The administrators can complete a FYI questionnaire either in the form of Likert scale responses or in the form of multiple-choice responses based on which best describes the child’s social and communication behaviour. The FYI is currently used for research and developmental purposes only. Baranek and the team are continuously researching and upgrading FYI to be used for clinical purposes in the future. Moreover, to identify the reliability of the tool, parents are provided with another questionnaire (FYI-R) using open-ended questions to understand how well FYI has been able to discern infants with autistic behaviours from other developmental disorder cases and controls. The main problem of this strategy is it still does not provide a quantitative value for the validity of the tool [52].

### 2.2. Adolescents and Adults ASD Screening Methods

#### The Ritvo Autism Asperger Diagnostic Scale- Revised (RAADS-R)

The RAADS-R is the latest and only self-scoring ASD screening tool developed for only adults (18 +). Created by Dr. Ariella Riva Ritvo in 2011, the intention was to identify undiagnosed adults with ASD and related social and communication disorders. The initial version of RAADS consists of a 78-item questionnaire covering three areas of neurodevelopment including language, social relatedness, and sensory motor. The RAADS-R is the revised version that covers an additional area of development—circumscribed interest [53] The RAADS-R version has 80 item instruments, including 78 items from the initial version. The RAADS is available online and takes approximately 20 min to complete the screening. Each item has four different parts, with different score values ranging from 0–3. These are ”true now and when I was young”, ”true only now”, ”true only when I was younger than 16”, and ”never true”. At the end of each screening, an overall score is generated based on the answers given to the questions. The users who gain an overall score below 65 are identified as the controls and the users who score above 65 are directed to further evaluations and clinical diagnosis for ASD. Further, each subscale of items is given a threshold score and anyone scoring above the threshold poses a risk of having ASD or other related disorder. The RAADS-R is considered to be an acceptable ASD screening instrument with 97% sensitivity and 100% specificity according to [53]. Further studies carried out by the authors on the validity of RAADS-R, using 779 samples, confirmed that only 3 users out of 100 receive false results from this tool, meaning that the tool generated 97% accurate information [53].

### 2.3. Hybrid ASD Screening Methods

#### 2.3.1. Asperger Syndrome Diagnostic Scale (ASDS)

Differentiating cases of Asperger syndrome is an absolute quandary due to the similarity of symptoms with autism and other developmental disorders. To traverse this gap, [54] have developed a quick, user-friendly, rating scale known as ASDS to differentiate cases of Asperger syndrome from other related disorders. The ASDS consists of 50 Yes/No questions that are designed to be administered by parents, caregivers, teachers, professional healthcare personnel, pathologists, psychologists, and specialists who are familiar with the child or adolescent who is being tested. The ASDS takes five specific areas of behaviour into consideration to identify Asperger syndrome in children and youngsters aged between 5 and 18 years: Cognitive behaviour, maladaptive behaviour, language, social and sensory, and motor behaviours. It usually takes 10–15 min to complete one questionnaire. Once the questionnaire has been completed, ASDS provides Asperger syndrome quotients (ASQ) that explain the individual’s likelihood of having Asperger syndrome. A high ASQ score indicates an increased probability of having Asperger syndrome and the need for further evaluations and clinical diagnosis. Meanwhile, a low ASQ score indicates controls of Asperger syndrome [54]. The sensitivity and specificity for the ASDS is unknown. However, the internal consistency of ASQ is reported to be 0.83, which is lower than the accepted level. Regardless, in a screening carried out using ASDS on 177 individuals with a wide range of neurodevelopmental disorders, 85% of them were correctly identified as cases of Asperger syndrome [55].

#### 2.3.2. Gilliam Asperger’s Disorder Scale (GADS)

The GADS was developed by [56] an associate professor at the University of Texas, to differentiate children, adolescents, and young adults with Asperger syndrome from those with autism and other developmental disorders. The GADS consists of 32 simple, clear, items designed to carefully align with the latest definition of Asperger disorder. Eight additional items are also included to obtain data from parents and caregivers on their child’s behaviour and progress over a three-year period. Later, in 2006, an expanded version of GADS, GADS-2, was introduced with 42 item instruments [56]. Both GADS and GADS-2 are designed to be used on anyone aged from 3 to 22 years, and should be completed by parents, teachers, or any other professional who is familiar with the patient’s behaviour. Once the items are completed, GADS generates a valid report that can assist in the proper medical diagnosis. The overall item score and percentile is calculated based on the summation of row scores for four standard subscales given for each item. The Asperger’s Disorder Quotient (ADQ), is used to predict one’s likelihood of having Asperger syndrome. A table of different ADQ values is provided for determining the need for further evaluation and diagnosis of Asperger behaviours. The validity of GADS is confirmed by its item’s conformity to the current clinical definition of Asperger syndrome, the robust interrelationship between each item, and how well it has been able to distinguish between patients with Asperger syndrome and other autistic behaviours. Even though widely used by many parties, and accepted as having a higher validity than the other ASD tools available for identifying Asperger disorder, the specificity and sensitivity of GADS is still unknown [57].

#### 2.3.3. Autism Spectrum Screening Questionnaire (ASSQ)

The ASSQ is also a hybrid screening questionnaire developed by [58,59] to identify the characteristics of high functioning developmental disorders, including autism and Asperger syndrome. It has 27 different questions, with three possible rating responses ranging from 0–2 as yes, somewhat, and no. The questionnaire is administrated by the parents or the teachers of the 7 to 16-year-old child or adolescent, and the result is interpreted based on the overall score gained by the individual. An overall score exceeding the threshold indicates that the individual has many characteristics of the autism spectrum and other high functioning syndromes. An accuracy of 90% is shown in the accurately attributed positive results generated by the tool at a cut-off threshold of 13 [44]. A recent study performed to validate the ASSQ screening tool reported that it has 91% sensitivity and 86% specificity [60].

#### 2.3.4. Autism Spectrum Quotient (AQ)

The AQ is a self-administrated ASD screening tool developed [20] along with other behavioural scientists from the Autism Research Centre, University of Cambridge, for identifying autism and other neurodevelopmental symptoms in adults with an average level of intelligence. The AQ questionnaire consists of 50 different questions covering the areas of social skills, attention switching, imagination, communication and attention to detail. The AQ test is available online and each question has four possible rating responses (definitely agree, slightly agree, slightly disagree, and definitely disagree) depending on which final score is calculated. The final score can range from 0–50 and a higher score indicates an increased level of autistic symptoms. A recent study on the validity of the AQ suggested that a cut-off score of 32 would optimise the validity of screening for adults in a clinical setting [20]. Later, in 2006 and 2008, two different versions of AQ were launched to cover adolescents and children [3,61,62]. AQ-Child is a parent-administered questionnaire specially designed for children aged 4–11 years whereas AQ adolescent is designed for teenagers aged 12–15 years. All versions of AQ contain 50 unique items and take approximately 20–30 min to complete.

To make it simpler and less time-consuming, [3], presented a compressed version of the original AQ adult version known as AQ-10-adult. Even though AQ-10 is shorter than the original version it has a predictive power similar to the original AQ version. The questions of QA-10 also have four possible responses, definitely agree, slightly agree, slightly disagree, and definitely disagree. The screening rule often considers one point per question. That is to say a point is assigned if the answer is either slightly agree or definitely agree for questions 1, 7, 8, and 10. In addition, a point is added if the user’s responses to questions 2, 3, 4, 5, 6, and 9 are either slightly or definitely disagree. The overall score is then calculated using a handcrafted diagnosis rule and anyone who scores above the threshold of six is considered to have autism and other related impairments. Lastly, [61,62], have developed full AQ versions for adolescents and children respectively. Ref. [3], then proposed shorter versions for the full adolescent and children’s AQ tests. The score calculations for the adolescent and child short versions are different from the AQ adult short version. The details of the questions, and the scoring of the adolescent and children AQ short versions, can be found in [3]. 

In terms of validity, AQ-child has the highest sensitivity (95%) and specificity (95%) out of all the versions of AQ. However, overall sensitivity and specificity of AQ are reported as 77% and 74% respectively at a cut-off score of 32 [62]. 

#### 2.3.5. Developmental Behaviour Checklist-Autism Screening Algorithm (DBC-ASA)

The DBC-ASA was derived from a version of DBC-P, the parent or primary caregiver checklist (as described under Developmental Behaviour Checklist—Early Screen (DBD-ES)) [42]. This version of DBC consists of a 29-item questionnaire that is intended to differentiate between young children aged between 4 and 18 years with ASD and those with other pervasive developmental disorders. The scores are calculated in the same way that is calculated in the DBC-P version, and an optimum level of sensitivity and specificity was found at an un-weighted cut-off score of 17 [63].

#### 2.3.6. Krug Asperger Disorder Index (KADI)

The Krug Asperger Disorder Index [64] is a screening instrument developed with the intention of identifying cases of Asperger syndrome and for setting goals for further medical interventions and individual educational programmes (IEP). The KADI covers two large segments of the population, including children from 6 to 12 years of age and adolescents and young adults aged between 12 and 21 years of age. The KADI screens individuals through a two-stage process, where the initial stage is called pre-screening. The individuals who are identified as positive for ASD are then directed to the second stage where the individual answers a parent report questionnaire consisting of 32 different items. The scoring and administration procedures of the KADI questionnaire are well defined and easy to follow. All 32 items are given two possible responses with two different weighted values respectively, that are related to the item description. The parent is instructed to read all the items carefully and circle the response that explains their child’s behaviour. The responses for the first 11 items are evaluated to identify whether the individual meets the basic criteria for a diagnosis of Asperger syndrome. If the basic criteria are not met, individuals are considered to be the controls and no further evaluation is needed. The individuals who meet the basic criteria then need to complete all 32 items and the final result is generated based on their overall score. The KADI is a widely accepted tool, as it distinguishes individuals with Asperger syndrome from those who do not have the syndrome, and has been able to achieve a sensitivity of 78% and specificity of 91% [65].

#### 2.3.7. Social Communication Questionnaire (SCQ)

The Social Communication Questionnaire is a clinically used ASD screening tool developed by three neuroscience physiologists to evaluate communication and social behavioural impairments of children that may be symptomatic of ASD. The questionnaire consists of 40 Yes/No questions that need to be administered by parents and primary caregivers of the child, and takes approximately 15 min to complete one screening process [66]. The SCQ is available in two basic formats: Lifetime and current. The lifetime version looks at the entire developmental history of the child whereas the current version focuses on the child’s behaviour only during the past three months. The results generated by both versions are subject to a cut-off score, where individuals who score beyond the cut-off score in the lifetime version are identified as possible autism cases and directed to complete further evaluations. The results generated from the current version are used to set goals for individual education programmes, further medical intervention, and treatment planning. An analysis was carried out on the ASQ tool to evaluate its validity and the degree to which the tool can discern individuals with autistic behaviours from other neurodevelopment disorders. The analysis suggested that SCQ has a sensitivity of 96% and specificity 80% at a cut-off score of 15. It also revealed that a higher cut-off is required to differentiate autism from other developmental disorders. Therefore, at the optimum cut-off score of 22, SCQ achieves a sensitivity of 75% and specificity of 60% [66].

#### 2.3.8. Social Responsiveness Scale (SRS)

The SRS is a family reported screening tool developed by [12], an Associate Professor in Psychiatry and Paediatrics at the University of Washington, which also aims to differentiate cases of ASD from other neurodevelopmental disorders. The SRS questionnaire consists of 65 items, with three possible Likert scale responses ranging from 0 to 3 (not true, sometimes true, often true, almost always true) and takes approximately 15–20 min to complete [12]. The overall scale is generated based on the responses given by the individuals, covering a large population of children and adolescents aged between 4 and 18 years. The SRS questionnaire can be administered by parents, relatives, spouses, or any other party who is familiar with the individual and is an acceptably valid tool for addressing the social responsiveness and related behavioural impairments of a wide range of individuals.

The SRS-2, an updated version of SRS, was launched in 2012 to address other areas of development such as the communication, interpersonal, and stereotype behavioural condition of ASD [67]. The SRS-2 allows individuals to use more than one examiner who is familiar with the rated individual and poses an average reading ability. The SRS-2 has four basic forms of instruments covering three age groups, each consisting of 65 different items. The pre-school form covers children aged 2½ to 4½ years and the school form covers children and teenagers aged between 4 and 16 years of age. The original SRS is used as the school form with no changes and can be administered by the parents or teachers of the child. There are two versions of the adult form. One allows self-administration while the other allows administration by parents, friends, spouses, or any other relative of the individual. Both adult versions cover a large population from age 19 to 89 years. 

The scoring of SRS-2 involves five subscales, including social awareness, social motivation, social cognition, restricted interests, and social communication. The social awareness subscale consists of eight items and evaluates the individual’s ability to understand social clues. The repetitive behaviour and restricted interest subscales have 12 items that address the stereotype and constrained behaviours of the individual. Social motivation measures the social interaction of the individual with 11 items, whereas social cognition uses 12 items to understand and interpret the social behaviour. The social communication subscale consists of 22 items and measures the ability for mutual communication. The end results are interpreted using the T-score, derived from the overall score of all 65 items. A T-score above 76 indicates the possibility of severe ASD diagnosis, whereas a T-score ranging from 66 to 75 and 60 to 65 indicates moderate and mild conditions of ASD respectively. Further, individuals with a T-score below 59 are considered the controls, so do not necessitate ASD clinical diagnosis [68].

However, SRS’s validity and reliability are clinically proven, and the studies have shown that it has a sensitivity of 78% and specificity of 94% at a cut-off score of 70 [69].

#### 2.3.9. Movement Assessment Battery for Children (25) (MABC-2)

The second edition of Movement ABC was developed by the Psychometric Centre of the UK with the collaboration of [23], founder of the initial version of Movement ABC. The MABC-2 is a norm-based screening tool established with the aim of evaluating the movement skills and disabilities of children and adolescents aged between 3 and 17 years. The MABC-2 consists of three motor tests designed for three age groups (3–6, 7–10, 11–17), and is twofold, as A and B where A focuses on evaluation of the child’s behaviour in a static, predictable, environment and B focuses on evaluating the child’s motor behaviour in a dynamic, unpredictable, environment. The number of items in MABC is reduced to 30 in MABC-2, and should be administered by the parents or an adult who knows the child well. The MABC-2 involves evaluating the emotional and motivational problems associated with the child’s motor skills. Each is further subdivided into three scales: Ball skills, dynamic and static skills, and self-care skills, consisting of five different items respectively. Each item is given four possible ratings, ranging from 0–3 (0 = very well, 3 = not close). The final decision is made by using the total motor score (TMS), derived from the overall score obtained by the child. The children whose score is above 95 are highly vulnerable to motor impairments in their daily life and those who score between 85 and 94 are at risk of being exposed to movement difficulties. The children whose score is below 85 are not considered cases of motor impairment and no further evaluations are required [23]. The main problem of MABC-2 is the lack of validity, as MABC-2 does not have an acceptable sensitivity (41%), even though the specificity is at a considerable level (88%) [70].

#### 2.3.10. Parents Evaluation of Developmental Status (PEDS)

The PEDS is the only evidence-based screening tool available to address parents’ concerns about the developmental and behavioural impairments of their children. It was developed by Frances Page Glascoe in 1996 while a Professor of Paediatrics at Vanderbilt University, USA [71]. The PEDS questionnaire consists of 10 items covering the following areas of development: Cognitive behaviour; expressive language and receptive language skills; fine motor and gross motor skills; behaviour; social and emotional behaviour; self-assistance; school behaviour, and others. The questionnaire is administered by the parents of children from 3 to 19 years of age and each item has three possible responses, ‘yes, a little, and no, with a separate space to mention the parents’ comments. The parents are required to circle the corresponding response and it does not take more than 10 min to complete one questionnaire. Once the questionnaire is completed, the scoring can be calculated by the administrator by downloading a scoring guide which is freely available on the internet. However, it is recommended that the PEDS score sheet be completed by a professional practitioner. The administrator is required to identify both predictable and none predictable impairments of the child and to indicate them in the appropriate columns of the score sheet. Finally, the score sheet is interpreted by the administrator and children are directed to appropriate pathways (A, B, C). The children who are directed to Path A are identified as at a higher risk of developmental disorders whereas Path B children are identified as cases with moderate risk. Even though path C indicates a low risk, children directed to path C need to be further evaluated for emotional and behavioural disabilities [71]. The PEDS is a well-performing tool for identifying various developmental and behavioural disorders with an average sensitivity of 79% and specificity of 80% [72].

#### 2.3.11. Australian Scale for Asperger Syndrome (ASAS) 

Ref. [73] developed an Australian Scale for Asperger Syndrome (ASAS) to identify Asperger behaviours in school children aged 6 to 12 years. The ASAS is administered by a clinician and the questionnaire consists of 25 items separated into five subscales: social and emotional abilities, communication skills, cognitive skills, specific interests, and motor skills. Each item is provided with seven possible rating responses, from 0–6, where 0 indicates the behaviours and abilities that are common in a normal child at that age and both 1 and 6 indicate rare and frequent behaviours respectively. However, the ratings between 2 and 6 do not always imply that the child is exposed to Asperger syndrome [73]. The accuracy and validity of the results generated by ASAS is reported to be as low as 65%, and there is no indication of the sensitivity and specificity. Therefore, the developers recommend that the tool should only be used as a screening instrument, not for clinical [74]. 

#### 2.3.12. Child Behaviour Checklist (CBCL)

The Child Behaviour Checklist is one of the oldest screening tools, developed in 1991 as a component of the Achenbach System of Empirically Based Assessments (ASEBA) established by Tomas Achenbach with the aim of identifying behavioural disorders of children and adolescents aged between 6 and 18 years [75]. There are two basic versions of CBCL: A preschool version and a school age version. The preschool version covers children aged from one and half years to five years and is administered by the parents or primary caregivers who interact with the child on a daily basis. The questionnaire consists of 100 questions with three possible Likert scale responses ranging from 0–2, where 0 indicates not true and 2 indicates very true. The school age version covers children and teenagers aged between 6 and 18 years, and the questionnaire consists of 118 items with rating responses similar to the preschool version. Eight important areas of the child’s development are screened through the questionnaires: Attention problems, aggressive behaviour, anxiety levels, rule breaking behaviour, social problems, somatic complaints, depression levels, and thought problems. Each represents one subscale on the checklist. Two different scores are derived from the eight subscales: Internalising problems and externalising problems. The summation of both internalising and externalising of problems presents the total problem score that is used to interpret the child’s behaviour. A higher total problem score indicates the higher risk of behavioural impairment. According to an evaluating study carried out on the validity of CBCL using a Brazilian sample population, it was revealed that the CBSL Brazilian version has a high sensitivity and specificity in identifying cases of behavioural disorders [76].

### 2.4. Comparison 

There is an increasing trend in studies related to autism symptoms and screening over the last three decades. Identified above are the common ASD screening tools that require the involvement of many stakeholders, such as parents, children, caregivers, patients, clinicians, psychologists, and other healthcare professionals, in taking the screening tests, calculating scores, explaining and interpreting tests results, and sometimes in processing further referrals. This section presents a comparison of identified tools in terms of their target audience, screening method, number of items, time consumption, and performance. The best performing tools are identified and requirements for further developments are discussed. Later, in Section 3, more in depth discussion is conducted with respect to different primary criteria.

The study evaluated different screening methods, some with multiple versions which brings the total to 37 ASD screening instruments that have been developed by various scholars. One of the oldest tools, CBCL was developed in 1991, and versions of AQ-10 (Adults, Child, and Adolescent) and Q-CHAT are recent screening tools having been developed in the year 2012. Among all the identified ASD screening tools, most focus on a specific range of the population, in particular infants and toddlers aged between 6 and 48 months. Less tools are available for adults aged 18 or more years. Many of the screening methods utilised one or more questionnaires to identify autistic behaviours, except for tools like MABC-2 and STAT which use play activities and physical observations to discriminate cases of autism from other related disorders. CBSL includes a questionnaire with 118 items, and thus has the maximum number of items, whereas the Child, Adolescents, and Adult short versions or AQ-10s and Q-CHAT-10 have the minimum number of items at only 10. Therefore, AQ-CHAT and AQ-10s are efficient methods since they take only 5–10 min to complete, whereas methods like CBCL and RAADS-R usually require much longer time to complete. In terms of sensitivity and specificity, CAST and ASIEP-3 have the highest sensitivity at 100% whereas CHAT and MABC-2 have the lowest sensitivities at 40% and 41% respectively. Similarly, RAADS-R has shown the highest specificity of 100% and DBD-ES has displayed the lowest recorded specificity at 48%.

Table 1 displays a comparison between the ASD screening tools available for toddlers and children aged between 6 months and 13 years. The CHAT is the oldest method, developed in 1992, whereas Q-CHAT-10 is the most recent. Most of the methods have questionnaires with 10 to 70 items related to ASD behaviour, communication, and social conditions. The STAT and ASIEP-3 are the only methods to use different activities to evaluate children’s behaviour. Most of the screening methods take approximately 10–25 min to complete, except ABC which takes more than 25 min. The CHAT and Q-CHAT-10 are more efficient than the other methods in terms of time required taking the test. The ABC, CARS-2, and ASEIP-3 are the most comprehensive methods available for screening individuals for autistic behaviours, as they cover a wider audience than the other available methods. Most of the ASD screening methods are not freely available on the internet and have limited accessibility. The Q-CHAT, CARS, CAST, and CSBC-DP are comparatively more accessible than the other methods and are freely available on the internet for anyone to download. The limited ASD screening smart phone applications are available for infants, toddlers, and children. In terms of validity, almost all the screening methods have acceptable sensitivity rates, ranging from 70–100%, and specificity between 80% and 100%. The CHAT’s initial version, CAST, and DBD-ES are the only ASD screening methods to have low sensitivities and specificities.

One of the few ASD screening tools involved in evaluating only adults is the RAADS-R (see Table 2). While many screening methods are designed for evaluating and discriminating autistic behaviours in adults, such as AQ, they include other age categories and thus are considered hybrids in this article. The RAADS-R is uniquely designed to identify autism and other related developmental disorders in adults over the age of 18. It is a valid and widely accepted screening tool that is freely available on the internet and maintains adequate levels of sensitivity and specificity.

Table 3 shows a comparison between screening methods in the hybrid category. These screening tools address developmental issues in infants, children, adolescents, and adults simultaneously by either employing the same screening tool for all, a two or more combination of the above age categories, or through employing different versions customised for each category of audience. The KADI screening tool uses one method to evaluate all children, adolescents, and adults for example, whereas AQ utilises different versions (short and full) to evaluate the behaviours of children, adolescents, or adults.

Almost all of the hybrid ASD screening tools utilise questionnaires as the method of evaluation regardless of their target audience, except for MABC-2 a special screening tool designed to identify motor impairments in children and adolescents. The MABC-2 is a unique screening tool that requires physical observation and interaction with the child to pre-determine their motor skills related disabilities. The tools that utilise questionnaires often consist of 10–70 items, with the exception of CBCL which includes 118 items. The tools such as the AQ short versions and PEDS are more efficient than the others, with only 10 items to answer and requiring far less time to complete (5–15 min). Further, AQ is more comprehensive and accessible than the other tools as it has several versions that cover many age segments and is freely available on the internet as both web-based questionnaires and smart phone apps. The AQ does not require professionals or specially trained individuals to administer the questionnaires, further increasing its accessibility. All the screening tools listed under the hybrid category are acceptable in terms of their sensitivity and specificity, except for MABC-2 which has the lowest sensitivity at 41%.

## 3. Discussion 

The section below is focused on evaluating the screening tools presented above in terms of their administration methods, accessibility, popularity, performance, and comprehensibility in order to shed light on the possible innovations required in new screening tools to be developed for screening autism. 

### 3.1. DSM-IV versus DSM-5 Criteria 

Many scholars continue to argue over the shortcomings of both CJ and self-reported screening tools, especially regarding efficiency and administration requirements [15,16,77,78,79]. The validity and reliability of most of the screening tools is still under investigation as most of them follow the earlier version of the DSM (*DSM-IV*) rather than the procedures and guidelines of the current *DSM-5* manual. Since most of the screening methods utilise different behavioural characteristics in determining a patient’s developmental age, they have been jointly presented as the triangle of impairments under the definition of the *DSM-IV* and still need to consider amendments presented in the *DSM-5* [80]. The most recent version of the DSM (*DSM-5*) groups the five PDDs, consisting of Asperger syndrome (AS), pervasive development disorder–not otherwise specified (PDD-NOS), Rett syndrome (RS), and childhood disintegrative disorder (CDD), into ASD [80]. The guidelines set by the *DSM-IV* are followed mostly by clinicians and healthcare professionals all around the world when diagnosing autistic behaviours. In the USA, the 10th version of the International Classification of Disease (ICD-10) is also used in diagnosis and clinical evaluations of autism and other developmental disorders [81]. The ICD-10 lists seven syndromes under PDD, and includes atypical autism and unspecified PPDs beyond the five PDDs listed by *DSM-5*.

Conventional methods used in clinical judgements (CJ) of ASD, such as ADI-R and ADOS, diagnose individuals based purely on behavioural criteria through a questionnaire or interview that contains items related to the *DSM-IV* [10,11]. After publication of the *DSM-5*, researchers pointed out that some cases who were diagnosed with autism using *DSM-IV* criteria may not be classified as having ASD under the revised *DMS-5* criteria [4,82,83,84]. This has created a debate among scholars in behavioural science, psychiatry, and psychology due to the inconsistent sensitivity and specificity results published in the last few years. For instance, [82] showed a reduction in sensitivity for adults and toddlers while [85], revealed a consistent sensitivity of cases tested under both the *DSM-IV* and *DSM-5* despite a decrement in specificity. 

Since most of the ASD screening methods available today were developed prior to 2013, they did not consider the guidelines established in the *DSM-5*. The existing ASD screening methods are based on clinical diagnosis methods, and therefore changes in ASD diagnosis criteria after publication of the *DSM-5* demanded a change in the way diagnostic algorithms within the screening method behaved during the classifying of cases. For instance, items in the existing screening methods should cover social interaction and social communication (Category A) in the *DSM-5* manual and at least two criteria from Category B (Restricted and Reparative Behaviour). Unfortunately, despite items in the majority of current screening methods fulfilling multiple criteria in Category A, they still fail to fully cover conditions in Category B. Nevertheless, screening for ASD does not necessarily require fully meeting the diagnostic conditions of ASD as its ultimate aim is merely to reveal potential autistic traits rather than diagnose individuals since to do so necessitates the involvement of expert clinicians and a clinical setup. 

Therefore, there is a need to re-examine questions and features within the ASD diagnostic and screening tools in order to comprehensively satisfy the new criteria of the *DSM-5*. This necessitates mapping the new ASD criteria to the items used in the screening tool besides evaluating the way the diagnostic process works. The outcome may result in an updated version of the current screening tool that maps the new criteria of ASD in the *DSM-5* to the items of the screening tool. In addition, comprehensive experimental studies using controls and cases as data are expected to be conducted in order to direct researchers, clinicians, psychiatrists, and psychologists to the right screening tool that maintains performance even after the new changes proposed. 

### 3.2. Digital Presence and Accessibility 

Some of the discussed ASD screening tools are available on the internet, either as web-based online tests or smartphone applications. Other instruments require a payment and are available only in hand written formats. These tools are intended to enhance development of disease control and prevention measures through early detection of ASD and associated communicational and behavioural disorders. The ASD screening tools, such as Q-CHAT, ASDS, ASSQ, CARS, AQ, CAST, PEDS, and CSBS-DP, are freely available on their web pages for access by parents, teachers, professionals, and clinicians who can administer the tests online and then receive an automatically generated score at the end of each test completed. Most web-based screening tools provide a guide to interpreting the final scores. Some screening tools like STAT, however, consist of a tool kit that includes a user manual, check list, questionnaire, score calculation manual, and sometimes toys such as dolls, balls, trucks, etc., in order to physically observe the child or rate an individual’s behaviour in detail. These types of screening tools are difficult to integrate into a mobile platform and require special training to administer. 

There are only a few screening tools available as mobile applications, and most of these use two or more combinations of the screening methods above to derive their results. Therefore, it is quite difficult to evaluate the methods used in smart phone apps in terms of sensitivity and specificity. The M-CHAT, AQ, and CBSL are the screening methods most commonly used in mobile platforms (Android, iTunes). However, a new mobile screening application based on all AQ short versions (AQ-10-Adult, AQ-10-Adolescent, AQ-Child) and Q-CHAT (toddlers) was recently developed to cover all age categories [86]. This is the only screening application available for all audiences. The Autism Fingerprint is another example of a smart phone application that uses M-CHAT as its screening method. In Oman, 14 out of every 100,000 children have been found to be cases of autistic traits, but there has been a lack of awareness and properly standardised tools to diagnosis the disease in the early stages. For this reason, Autism Fingerprint was developed by Arab neuroscience specialists in collaboration with technical experts [87]. Culturally and traditionally appropriate images and items were used in order to make the application more user-friendly and an easy to use tool for screening children for autistic traits. The AQ Asperger Test, AQ Test, and the Asperger Test are some of the mobile applications that were developed using AQ. These applications can be found in both Android and iTunes mobile platforms. The Canvas Child Behaviour Check List is such a mobile application, using CBCL as the screening method to investigate behavioural impairments in children and adolescents aged between 4 and18 years.

There are many other applications that use games, drawing tools, and advanced online activities to observe the behavioural conditions of individuals, covering various segments of the population. Apple iPad is considered one such advanced tool that helps children with special needs in their communication and social development programmes. The specialists in the world’s health care arena have advised that since the iPad came onto the market in 2010, it has helped many children with autistic behaviours in developing their skills [88]. Similarly, the contribution of numerous and innovative applications introduced by Android for promoting awareness and identifying individuals with autism at an early stage has been immense.

The presence on the internet and mobile platforms is essential in today’s society to ensure the accessibility of any product or service. Therefore, the availability of free ASD screening tools via the internet defines their accessibility. The accessibility and comprehensiveness of some screening tools is questionable, however, as most are not freely available and only target specific demographic groups. Even though some of the ASD screening tools are available on web platforms, they are not free for users. Most of the tools available on the internet are subject to a certain payment prior to obtaining access to the screening process. The ASD screening instruments, such as the AQ versions, Q-CHAT, and CAST are freely available for anyone to use while tools such as CARS-2 and CSBS-DP are available on the internet as a pay only facility. In a world where the demand for smart phone applications is growing rapidly and even the most basic facilities are available in mobile application format, it is crucial for ASD screening tools to be present on mobile phone application platforms. Currently, only a few screening tools, such as AQ and CSBS-DP, are available on smart phone platforms. The unavailability on the internet and mobile platforms hinders the accessibility to users of many of the screening tools.

### 3.3. Administration and Time Efficiency

Administration refers to undertaking the questionnaires or interviews provided by the ASD screening tools in order to identify autistic behaviours and an individual’s likelihood of being a case of autism. There are three types of ASD screening tools: Self-administered, parent or caregiver administered, and administered by clinicians or well-trained professionals. Most of the screening methods discussed fall into either the self-administered or parents/caregiver-administered category. Some methods require professionals to administer the questionnaires and/or to score and interpret the generated results. This is one additional requirement that makes the screening tools hard for ordinary people to use. The Q-CHAT and STAT are examples of screening instruments that require administration by professionals in addition to a report submitted by the parents on the behavioural complexities of their child. Some screening tools, such as CSBS-DP, allow the parents, teachers, and other caregivers who are familiar with the individual to administer the questionnaire, but scoring and interpretation of the scores is required to be done by a professional. Thus, these screening tools have limited involvement expected of the potential users. On the other hand, the screening tools that are self-administrated, such as AQ and its versions, seem to have fewer requirements to be conducted and can be taken by adults with average IQ, parents, family members, caregivers, and teachers among others. These self-administered methods often utilise simple scoring functions that offer a numeric score for the likelihood of having autistic traits.

One of the key performance indicators of the ASD screening tools is the time taken to complete one screening process. Since conventional methods are usually lengthy questionnaires that take time to complete, and many of the screening tools have originated from these questionnaires, it is advantageous to reduce the time necessary for the test. For example, the AQ-adolescent version originally had a questionnaire with 50 items that took approximately 15–20 min to complete. Ref. [3] dealt with this shortcoming by reducing the number of items in the original AQ questionnaire to 10 items, only taking 5–8 min to complete. Many scholars have not yet recognised this need for a short and effective screening tool, and therefore have less involvement by users. RAADS-R, CSBS-DP, and KADI are some of the tools that take more than 30–45 min to complete, even though they are widely accepted in terms of their reliability and validity.

In the current digital era, most users prefer to have shorter screening tests such as Q-CHAT-10, AQ-10 versions, and PEDS since the tests are typically taken in an online environment and within an acceptable timeframe. In fact, recent developments in hardware, computer networks, and mobile applications have provided rapid accessibility to the tools for the healthcare community. New technologies, such as mobile platforms, may render some of these time-consuming tools obsolete. 

### 3.4. Performance and Comprehensibility 

The validity and reliability of the ASD screening tools are expressed in terms of sensitivity and specificity metrics when applied against a certain dataset of cases and controls. It is imperative, therefore, to acknowledge that these two metrics (besides accuracy) are measured with respect to a specific dataset. Thus, the screening method performance is restricted to the dataset characteristics and ensures quality. According to [89] sensitivity refers to the ability of the screening tool to identify a person who is a case of autism while specificity refers to the power of the screening tool to discriminate a person who is a control of autism. Based on the results reported in the literature (and included in the tables constructed in Section 2.4), most of the existing screening methods have acceptable sensitivity and specificity rates. Nevertheless, some screening tools have little research validating their results with respect to sensitivity and specificity metrics. For instance, the MABC-2 method, which has the least reported sensitivity (41%) and the ESAT method which has the least reported specificity (14%) are examples of screening tools that could potentially be improved to obtain acceptable levels of sensitivity and specificity on their datasets. The tools such as M-CHAT, FYI, GADS, and ASAS still necessitate experimental studies to seek their actual performance (both sensitivity and specificity).

The comprehensibility of the screening tools depends on the size of the audience that they cover. Most of the screening tools cover only one segment of the population, with some being specialised for infants and children while others are designed specifically for adults. Since the recognition of autism at an early stage is critical for medication and treatment planning, many tools cover infants and toddlers aged between 12 and 36 months. A lack of importance placed on teenagers and adults is another issue associated with existing ASD screening instruments. Even though the instruments, such as M-CHAT and Q-CHAT, are present on both web-based and mobile phone platforms, with acceptable levels of sensitivity and specificity, the comprehensibility of these tools is in question as they only cover infants aged from 16–30 months. This represents less than 5% of the entire population. 

### 3.5. Popularity

There is no exact metric for measuring the popularity of an ASD screening tool as no tracking is available to measure the number of individuals who use a screening tool at any given time, nor how frequently they are being used. An estimate on popularity can be derived from the clinical usage of each tool. Unfortunately, most available ASD screening tools are designed for research and developmental purposes rather than clinical diagnosis purposes, with only a few tools such as CARS-2HF, ADOS, and ADI-R being used by clinicians in their medical diagnosis process. None of these screening tools can be used alone to provide a proper medical diagnosis, and are used in collaboration with many other medical tests and professional investigations in order to reveal autistic behaviours and to differentiate them from related developmental impairments. 

Some of the ways to measure the popularity of a screening tool is to utilise application features like functionalities, user review ratings, and coverage. Out of the testing methods considered, the AQ short versions and Q-CHAT have been able to obtain positive ratings across approximately 100+ user reviews. For example, the ASDTests app, which is based on the AQ short versions, has 111 reviews and numerous downloads. Apparently, the screening tools that are based around questionnaires are more favourable to end users, as observation and video screening methods have limited or no ratings in both the Android and Apple stores. It is believed that this is the result of these screenings being more time consuming than questionnaire-based methods. In questionnaire tests, such as AQ-10, the number of questions is just 10 so users are less likely to lose interest while using this method. The hybrid screening methods seem to cover a larger group of users, as they target various combinations of toddlers, children, adolescents, and adults. More importantly, only one screening method application, ASDTests, covers all audiences collectively, thus making it more popular. This shows that hybrid screening methods seem to be more comprehensive than specific screening methods, at least in the context of user usage. Nevertheless, methods such as Q-CHAT, that cover toddlers, are still popular within their user segment (toddlers).

### 3.6. Intelligent Classification Methods

The current CJ and ASD screening tools generally employ human developed rules to classify cases and controls. The psychiatric and behavioural science specialists have designed these rules, and the quality of outcomes and decisions depends substantially on the subjective contributions of these professionals and the interpretations of the specialised clinical staff conducting the assessments. Instead, the diagnosis of ASD might be empowered by automated decisions generated by intelligent algorithms such as machine learning. To date, there are no self-administered ASD diagnostic methods that have integrated machine learning models into the process, despite a few research attempts on doing so [15,16,17,78,79,90,91,92,93,94] The lack of integrating technology with existing methods may contribute to current limitations. For example, the ASD classification amendments from 2013 were disseminated in an updated version of the *DSM-5*, but ASD diagnostic procedures were not changed in accordance with these amendments. The machine learning innovations to be examined and developed for self-assessment tools are intended to make the classification process of ASD automated, rather than static. These changes may effectively replace pre-existing human-generated rules and procedures, resulting in three distinct and impactful advantages: Increased efficiency with ASD classification (less time required for screening); the reduction in the number of questions and components of ASD assessments to minimal levels while maintaining assessment integrity and validity (identification of key components that produce accurate diagnoses); and the enhancement of classification accuracy for borderline and complex cases due to empowering predictive models derived by machine learning algorithms that facilitate ASD classification decisions. 

The self-administered components of the assessment, with respect to screening tools, is expected to be automated using machine learning and facilitated by caregivers or professionals. This necessitates the following: (1) The minimisation of the total number of scale items through computational intelligence techniques; (2) the creation of a machine learning classification algorithm to be embedded within the classification process; (3) examination of the case or control; (4) periodic amendments to machine learning algorithm outcomes (i.e., predictive models), based on the classified test cases.

It is also possible that the development of a new ASD self-administered assessment tool, based on machine learning, will encourage a transition from antiquated CJ tools and contribute to increased efficiency with professional diagnostic processes. Future directions with CJ tools may involve a semi-automated process, due to the need for licensed clinical specialists to verify outcomes (i.e., specific classifications). The sssessment conclusions will be solely in the hands of the specialists, while machine learning will continue to improve predictive models and provide applicable alternatives to professionals. Furthermore, machine learning may provide assessors with potential rationales for classification decisions, improving the diagnostic process with respect to both efficiency and accuracy.

## 4. Conclusions 

Autism is no longer a dilemma for ordinary people due to the rapidly increasing awareness of the ASD spectrum and availability of screening and diagnosis measures. Identifying and distinguishing autistic traits from other developmental disorders has not been simple and easy in the past. Consequently, many scholars in the behavioural science, psychology, psychiatry, and neuro-science fields have developed diagnosis and screening methods to identify cases of autism and assist the medical diagnosis. There have been a few reviews on screening methods that have addressed common criterion, such as the number of items included in each screening test, time taken to complete the test, age categories involved, and performance (sensitivity, predictve accuracy, and specificity). However, existing reviews have failed to critically analyse vital aspects related to ASD screening, including the tool’s accessibility, comprehensibility, popularity, and efficiency among others. More importantly, none of the reviews emphasise the importance of the *DSM-5* criteria for evaluating the reliability of ASD screening. Therefore, this study investigates ASD screening methods to identify their performance in terms of different advanced parameters in order to discover possible concerns that need to be addressed through an innovative ASD screening process. A total of 37 different screening methods have been identified and categorised into three subcategories depending on their target audience in order to make the evaluation process more convenient. The three subcategories are: screening tools for infants and children, screening tools for adolescents and adults, and hybrid screening tools. Hybrid screening refers to the ASD screening tools that consider the target audience as a combination of three or more of the following categories: infants, toddlers, adolescents, and adults. Out of the 37 screening methods considered, 12 fall into the category of infants and children. All screening tools have been critically analysed individually in terms of their evaluation, administration, target audience, scoring methods, other available versions, and performance. None of the prevailing screening tools have been found to be performing completely well in terms of all the considered parameters. Some tools that were apparently performing well in terms of their sensitivities and specificities have been found to be unsuccessful in other parameters. For instance, ASIEP-3 is a highly accepted tool with a 100% sensitivity and acceptable level of specificity (81%). However, it consists of a series of activities and a questionnaire with 47 items, making it more time consuming than the other available tools. Similarly, CHAT is a tool that is efficient in terms of time, but is not freely available and has an unacceptable level of sensitivity (40%).

Many of the available screening tools, especially the short versions, comply only partly with the ASD criteria of the *DSM-5* introduced in 2013. Most of the available tools were developed before that time and follow the guidelines established by the older version, *DSM-IV*. Apart from that, each screening tool has been discussed in depth in terms of accessibility, comprehensibility, administration, popularity, and performance. It has been revealed that many available screening tools, such as Q-CHAT and STAT, require administration by well-trained professionals (at least during one stage of the evaluation). Moreover, the M-CHAT and Q-CHAT are not comprehensive in terms of the size of the audience they cover, but for an individual who is looking for a screening tool to identify autistic traits in an infant aged from 16 to 36 months these are appropriate methods as they perform well in terms of sensitivity and specificity. Similarly, the AQ-10 (Adults and Adolescents) can be recommended for individuals aged 12–16 years and 18+ respectively, as they are time efficient, easy to use, and can be self-administered with an acceptable level of performance.

With the exception of AQ, AQ-10, Q-CHAT, CARS, CAST and their variations, many of the tools are not freely available for users and only three tools are available on a mobile platform, thus limiting accessibility for users. Some screening tools also have issues with their performance and comprehensibility, especially the lengthy time questionnaires (some with more than 50 items). This makes the entire screening process tedious, unpopular, and not very usable by individuals. To summarise, the findings of this study emphasises the need for a more efficient, intelligent, and innovative ASD screening tool that can cover a wider audience while maintaining high levels of performance. 

It is believed that in the near future a highly interactive platform, utilising an intelligent machine learning diagnosis algorithm, will offer more accurate and robust performance that engages individuals with ASD (both children and adolescents), parents, caregivers, GPs, other medical staff, researchers, and the broader population. This is due to such intelligent screening methods being useful by offering both the diagnosis and the individual development plan needed for cases to their families and healthcare providers efficiently.

## Figures and Tables

**Table 1:** Summary of screening methods available for infants and children.

METHOD	TYPE	FEES	ITEMS	TARGET	AGE	TIME/MINS	SENSITIVITY	SPECIFICITY	WEB	MOBILE
CHAT (Checklist for Autism in Toddlers)	Questionnaire	N	14	Toddlers	18-24 months	8 to 15	40%	98%	X	X
M-CHAT & M-CHAT-RF (Modified Checklist for Autism in Toddlers & With Follow Up)	Questionnaire		23	Toddlers	16–30 months	10 to 20	95-97%	99%	X	X
Q-CHAT (Quantitative CHecklist for Autism in Toddler)	Questionnaire	N	25	Toddlers	18 to 24 months	15 to 20	88%	91%	Y	X
Q-CHAT-10	Questionnaire	N	10	Toddlers	19 to 24 months	5 to 10	91%	89%	Y	X
CAST (Childhood Asperger Syndrome Test)	Questionnaire	N	37	Children	5 to 11 years	15 to 25	100%	97%	Y	X
DBD-ES (Developmental Checklist-Early Screen)	Questionnaire	Y	17	Toddlers	18 to 48 months	10 to 15	83%	48%	X	X
ESAT (Early Screening for Autistic Traits)	Questionnaire		14	Toddlers	16 to 30 months	10 to 15	88%	14%	X	X
PDDST-II (Pervasive Developmental Disorders Screening Test-Second Edition)	Questionnaire	Y	22	Toddlers	18 to 48 months	10 to 20	92%	91%	X	X
ITC (Infant toddler checklist)	Questionnaire		24	Toddlers	6 to 24 months	10 to 15	89%	85%	X	X
FYI (The First Year Inventory)	Questionnaire		63	Toddlers	12 months	20 to 35	NA	NA	X	X
ASIEP-3 (autism screening Instrument for Educational Planning - Third Edition)	Questionnaire & Activities		47	Toddlers & children	2 to 13 years	Varies	100%	81%	X	X
CSBS-DP (Communication and Symbolic Behavior Scales Developmental Profile)	Questionnaire	Y	24	Toddlers	6 to 24 months	10 to 15	78%	84%	Y	X
STAT (Screening Tool for Autism in Toddlers and Young Children)	Play activities	Y	12	Toddlers	24 to 36 months	15 to 20	95%	73%	X	X
ESAT (Early Screening for Autistic Traits)	Questionnaire		14	Toddlers	16 to 30 months	10 to 15	88%	14%	X	X
(CARS)-2 Childhood Autism Rating Scale	Questionnaire	Y	15 X 2	Children	V1: < 6 years and V2: 6 to 13 years	10 to 20	81%	87%	Y	X
ABC (Autism Behavior Checklist)	Questionnaire	Y	57	Children	3 to 14 years	20 to 30	77%	91%	X	X

**Table 2:** Summary of the screening methods available for adolescents and adults.

METHOD	TYPE	ITEMS	TARGET	AGE	TIME/MINS	SENSITIVITY	SPECIFICITY	WEB	MOBILE
RAADS-R (The Ritvo autism Asperger diagnostic scalerevised)	Questionnaire	80	Adults	> 18	25 to 45	97%	100%	Y	X

**Table 3:** Summary of the hybrid Autism spectrum disorder (ASD) screening methods.

METHOD	TYPE	FEES	ITEMS	TARGET	AGE	TIME/MINS	SENSITIVITY	SPECIFICITY	WEB	MOBILE
ASDS (Asperger Syndrom Diagnostic Scale)	Questionnaire	Y	50	Children & Adoloscent	5 to 18 years	20 to 30	NA	NA	Y	X
ASSQ (Autism Spectrum Screening Questionnaire)	Questionnaire		27	Children & Adoloscent	7 to 16 years	10 to 15	91%	86%	Y	X
AQ(Autism Spectrum Quotient)	Questionnaire	N	50	Adult	> 18 years	20 to 30	93%	52%	Y	Y
AQ-10-Adult	Questionnaire	N	10	Adult	> 18 years	5 to 10	77%	74%	Y	Y
AQ-Adolescent	Questionnaire	N	50	Adolescent	12 to 15 years	20 to 30	NA	NA	Y	Y
AQ-Child	Questionnaire	N	50	Children	4 to 9 years	20 to 30	95%	95%	Y	X
AQ-10-Adolescent	Questionnaire	N	10	Adolescent	12 to 15 years	5 to 10	NA	NA	Y	X
AQ-10-Child	Questionnaire	N	10	Children	4 to 11 years	5 to 10	NA	NA	Y	X
DBC-ASA (Developmental Behavior Checklist-Autism Screening Algorithm)	Questionnaire		29	Children & Adoloscent	4 to 18 years	15 to 20	86%	69%	X	X
GADS (Gilliam Asperger’s Disorder Scale)	Questionnaire	Y	32	Children, Adoloscent and partly adults	3 to 22 years	15 to 25	NA	NA	X	X
GADS-2 (Gilliam Asperger’s Disorder Scale)	Questionnaire	Y	42	Children, Adoloscent and partly adults	3 to 22 years	15 to 25	NA	NA	X	X
KADI (Krug Asperger’s Disorder Index)	Questionnaire	Y	32	Children, Adoloscent and partly adults	6 to 22 years	21 to 35	78%	91%	X	X
SCQ (Social Communication Questionnaire)	Questionnaire		40	Children & Adoloscent	< 4	10 to 20	58-62%	93-100%	X	X
SRS (Social Responsiveness Scale)	Questionnaire		65	Children & Adoloscent	4 to 18 years	20 to 30	67%	78%	X	X
SRS-2 (Social Responsiveness Scale)	Questionnaire		65	Children & Adoloscent	4 to 18 years	20 to 30	78%	94%	X	X
PEDS (Parents Evaluation of Developmental Status)	Questionnaire		10	Toddlers & children	3 to 19 years	5 to 10	79%	80%	Y	X
CBCL (Child 28 Behavior Checklist)	Questionnaire		118	Children & Adoloscent	6 to 18 years	25 to 40	75%	82%	X	Y
